# Assessment of the performance of hidden Markov models for imputation in animal breeding

**DOI:** 10.1186/s12711-018-0416-8

**Published:** 2018-09-17

**Authors:** Andrew Whalen, Gregor Gorjanc, Roger Ros-Freixedes, John M. Hickey

**Affiliations:** The Roslin Institute and Royal (Dick) School of Veterinary Studies, The University of Edinburgh, Midlothian, Scotland, UK

## Abstract

**Background:**

In this paper, we review the performance of various hidden Markov model-based imputation methods in animal breeding populations. Traditionally, pedigree and heuristic-based imputation methods have been used for imputation in large animal populations due to their computational efficiency, scalability, and accuracy. Recent advances in the area of human genetics have increased the ability of probabilistic hidden Markov model methods to perform accurate phasing and imputation in large populations. These advances may enable these methods to be useful for routine use in large animal populations, particularly in populations where pedigree information is not readily available.

**Methods:**

To test the performance of hidden Markov model-based imputation, we evaluated the accuracy and computational cost of several methods in a series of simulated populations and a real animal population without using a pedigree. First, we tested single-step (diploid) imputation, which performs both phasing and imputation. Second, we tested pre-phasing followed by haploid imputation. Overall, we used four available diploid imputation methods (fastPHASE, Beagle v4.0, IMPUTE2, and MaCH), three phasing methods, (SHAPEIT2, HAPI-UR, and Eagle2), and three haploid imputation methods (IMPUTE2, Beagle v4.1, and Minimac3).

**Results:**

We found that performing pre-phasing and haploid imputation was faster and more accurate than diploid imputation. In particular, among all the methods tested, pre-phasing with Eagle2 or HAPI-UR and imputing with Minimac3 or IMPUTE2 gave the highest accuracies with both simulated and real data.

**Conclusions:**

The results of this study suggest that hidden Markov model-based imputation algorithms are an accurate and computationally feasible approach for performing imputation without a pedigree when pre-phasing and haploid imputation are used. Of the algorithms tested, the combination of Eagle2 and Minimac3 gave the highest accuracy across the simulated and real datasets.

## Background

In this paper, we review and analyse the use of imputation methods based on hidden Markov models (HMM) for animal breeding populations. Genotype imputation is a key aspect of many modern animal breeding programs and makes it possible to obtain genetic information on a large number of animals at a low cost. In breeding programs, a small subset of individuals (e.g., sires) are typically genotyped at high density and the remaining animals are genotyped at lower densities. Statistical regularities between shared chromosomal segments are used to impute the untyped loci. Modern methods impute missing genotypes at a very high accuracy [1–3], which increases the number of animals that can be genotyped for a fixed budget [4, 5]. The larger number of genotyped animals increases the accuracy of genetic predictions [6] and/or offers the potential to increase selection intensity [7, 8].

Traditionally, pedigree and heuristic imputation methods have dominated animal breeding [1, 9, 10]. These methods use large chromosome segments that are shared between closely-related animals to impute untyped or otherwise missing loci rapidly and accurately. In contrast, imputation methods used in human genetics have been based largely on the probabilistic HMM framework of Li and Stephens [11]. These probabilistic methods tend to have a higher accuracy than heuristic methods when used on datasets where individuals are not closely related or when pedigree information is not available. However, the computational cost of these methods has traditionally been too high for routine imputation in animal breeding populations.

In the last few years, the speed of HMM methods has improved, which allows the imputation of hundreds of thousands of individuals to hundreds of thousands of loci in reasonable computational time [12, 13]. These improvements have been driven by the widespread availability of large haplotype reference panels, and the emergence of a two-step imputation pipeline, in which observed genotypes are first phased and then untyped loci are imputed based on their phased haplotypes [14]. The improved scaling of HMM methods may allow for their routine use in large animal breeding populations. This progress is particularly timely, given the increasing interest in performing imputation in settings where pedigree information is not available, e.g. in developing countries or in non-nucleus commercial environments. Given the lack of appropriate public domain haplotype reference panels for many animal populations, smaller population sizes, and sparser marker densities, it is not clear whether these advances in HMM will be made for imputation in animal breeding populations. In addition, several HMM imputation methods have been developed and it is not clear which is most suited for routine use in animal breeding.

In this paper, we review several imputation methods and study their performance on simulated and real data. We grouped comparisons based on single-step (diploid) imputation methods and a two-step method that are based on a combination of pre-phasing and haploid imputation. Specifically, for diploid imputation, we evaluated fastPHASE [15], Beagle v4.0 [16], IMPUTE2 [17], and MaCH [18]. For pre-phasing, we evaluated SHAPEIT2 [19], HAPI-UR [20], and Eagle2 [12], followed by haploid imputation with IMPUTE2 [17], Beagle v4.1 [12], or Minimac3 [21]. First, we provide a review of these methods and then evaluate their performance on simulated and real data.

### Hidden Markov models

All of the methods considered here are based on the HMM framework of Li and Stephens [11], in which an individual’s genotype is modelled as a mosaic of haplotypes from a reference panel $H = \{ h_{1} \ldots h_{k} \}$. The methods calculate the probability that the individual has the pair of haplotypes, $h_{j}$ and $h_{k}$ at a locus $i$ given the observed genetic data, $G ,\;p(h_{ij} ,h_{ik} |G )$. To account for linkage between adjacent loci, the methods evaluate the probability of a haplotype, based on its fit to the observed genotypes at the loci and its similarity to the haplotypes inferred at nearby loci:

1 $$p\left( {h_{ij} ,h_{ik} |G } \right) = p\left( {h_{ij} ,h_{ik} |g_{i} } \right)p\left( {h_{ij} ,h_{ik} |h_{i - 1} ,h_{i + 1} } \right)p\left( {h_{i - 1} |g_{ - i} } \right)p\left( {h_{i + 1} |g_{ + i} } \right).$$

In this equation, the term $p(h_{ij} ,h_{ik} |g_{i} )$ measures the fit between the pair of haplotypes and the observed genotype at a locus; the term $p(h_{ij} ,h_{ik} |h_{i - 1} ,h_{i + 1} )$ captures transitions between haplotypes given the haplotypes at neighbouring loci; and the terms $p(h_{i - 1} |g_{ - i} )$ and $p(h_{i + 1} |g_{ + i} )$ measure the fit between haplotypes and observed genotypes at the remaining loci. These probabilities can be calculated using a standard forward–backward algorithm [22].

Traditionally, methods that rely on the framework of Li and Stephens [11] scale linearly with both the number of individuals and the number of loci, and quadratically with the number of reference haplotypes. The quadratic scaling is due to uncertain phase at heterozygous loci, which requires the methods to model haplotypes that are assigned on both chromosomes simultaneously. The quadratic scaling quickly leads to intractable computational costs, even for small reference panels, but this can be avoided if the low-density individuals are pre-phased, which allows haplotypes to be considered independently. As a result, haploid imputation, i.e. imputation with pre-phased haplotypes, scales linearly with the number of individuals, the number of loci, and the number of reference haplotypes.

In this paper, we consider two classes of HMM. In the first class, diploid imputation methods perform phasing and imputation simultaneously, which results in quadratic scaling with the reference panel size. To mitigate the latter, each of the evaluated methods, fastPHASE, Beagle v4.0, IMPUTE2, and MACH, apply their own strategy to reduce the effective number of reference haplotypes while maintaining high accuracy. In contrast, two-step imputation methods treat phasing and imputation as separate problems. Individuals are first phased and then imputed using a haploid HMM, which scales linearly with the number of reference haplotypes. Phasing methods may have either quadratic, super-linear, or linear dependence on the number of reference haplotypes. To increase phasing speed and accuracy, a number of techniques are deployed that could not be used if the phasing methods also had to handle genotype uncertainty at untyped loci.

Intuitively, we might expect that the diploid imputation methods will have higher accuracy (at a higher computational cost) than performing phasing and imputation separately because they automatically handle phase uncertainty. This is not necessarily the case if most errors in imputation stem from the inability to find appropriate reference haplotypes that would explain observed genotypes. By performing pre-phasing and then imputation, it may be possible to consider a much larger number of reference haplotypes and thus increase accuracy by finding a more appropriate set of reference haplotypes, which offset accuracy losses due to phasing errors.

Below, we review methods for diploid imputation, haploid imputation, and phasing.

### Diploid imputation

All four diploid imputation methods evaluated here use a haplotype state-space reduction technique to alleviate the impact of modelling a large number of reference haplotypes. IMPUTE2 and MaCH use subsampling, where the haplotypes considered in each iteration are a sample of the total haplotype pool. fastPHASE and Beagle v4.0 use haplotype clustering, where the overall number of haplotypes is collapsed into a smaller number of “ancestral” haplotypes.

Both IMPUTE2 and MaCH are run over a series of iterations. During an iteration, a subset of the haplotype reference panel is used to phase and impute each individual’s genotypes. In MaCH, the subset is selected randomly. In IMPUTE2, the subset is selected such that it is made up of haplotypes that are “nearby” the currently estimated haplotype for the individual. If these methods are run without an external reference panel, a reference panel is built up from the current phasing of high-density individuals. At each iteration, a new subset of the reference panel is selected for each individual, individuals are imputed and phased based on that subset, and then a reference panel is re-computed from the currently inferred haplotypes. The methods are run for a small number of iterations (e.g., 20) and the imputation results are averaged across iterations. There is a potential issue in applying these methods in populations of many closely-related individuals, due to the potential for feedback between the phasing of closely-related individuals [23].

In contrast, in fastPHASE and Beagle v4.0, individuals are imputed based on a set of estimated “ancestral” haplotypes. In fastPHASE, an expectation–maximisation algorithm is used to infer a small number of ancestral haplotypes from the data (e.g., 30) and the method then iterates between estimating the haplotypes of each individual as a mosaic of ancestral haplotypes and estimating the ancestral haplotypes based on the haplotype assignments for each individual. Beagle v4.0 uses a similar approach as fastPHASE, but instead of using a fixed number of ancestral haplotypes, it infers the number of ancestral haplotypes at each marker and models the transition between ancestral states at adjacent markers in the form of a directed acyclic graph.

### Haploid imputation

In contrast to the four diploid methods, haploid methods do not need to use a state-space reduction technique to handle moderate numbers of haplotypes, because they consider each phased chromosome independently and scale linearly with the number of haplotypes in the reference panels. However, with the recent focus on imputing large bio-bank size human populations (over 100,000 individuals) to whole-genome sequence level data, many of the current haploid methods use other techniques to reduce the computational burden when analysing large numbers of individuals at a large number of markers.

The haploid HMM used by Impute2 is a straightforward extension of the diploid method implemented in the same program and uses a subset of haplotypes (based on their similarity to the individual’s current phasing) to impute individuals. Minimac3 uses a similar technique, but instead of subsetting the reference panel, it uses a loss-less haplotype compression technique that combines haplotypes that are identical in a region and updates the likelihood of those haplotypes simultaneously. This update is particularly useful for whole-genome sequence data, which may display limited haplotype variation over long windows. Beagle v4.1 moves away from the graph-based haplotype model in Beagle v4.0 and uses the more traditional model of Li and Stephens. To reduce computational burden, Beagle v4.1 aggregates adjacent loci together into “strings” and performs updates based on “strings” instead of individual markers. In addition, it only updates the haplotype probabilities at genotyped loci and linearly interpolates the haplotype probabilities at untyped loci.

### Pre-phasing methods

Just as with diploid imputation, HMM-based phasing methods naively scale quadratically with the number of haplotypes in the reference panel. However, this quadratic scaling can be avoided by a state-space reduction technique of splitting the chromosomes into small windows and assuming that linkage information decays quickly across the window boundaries. Both SHAPEIT2 and HAPI-UR use a window-based approach, whereas Eagle2 manages the quadratic dependence by performing a limited beam search through the haplotype space.

SHAPEIT2 operates by splitting the chromosome into small haplotype windows, each containing three heterozygous loci. For each window, there are 2^3^ = 8 possible ways to phase it, and there are 2^6^ = 64 possible transitions between windows. SHAPEIT2 evaluates the probability of each of the eight possible haplotypes and 64 transitions based on a haplotype reference panel, and then phases individuals by sampling haplotypes based on their posterior probabilities. The probability of a haplotype in a given window and transition between windows can be evaluated in a time that scales linearly with the number of reference haplotypes. As in IMPUTE2, SHAPEIT2 subsets the haplotype reference panel by selecting haplotypes that are nearby the current haplotypes of the individual.

The window splitting approach may lead to lower imputation accuracy in breeding populations, where individuals are expected to share long chromosome segments. In SHAPEIT2, only the transmission probabilities between windows are modelled, not the probabilities of the underlying reference haplotypes. This means that haplotype assignment information from a given window is only used to update the next window and is ignored for further windows. This approach limits the amount of long-range haplotype information (covering more than three heterozygous loci) that can be exploited. One solution to this is to increase the window size.

HAPI-UR takes a similar approach to SHAPEIT2 in reducing the large state-space but uses a series of growing windows, which allow it to exploit longer shared chromosomal segments. In order to process large windows, HAPI-UR takes advantage of a number of computational techniques to reduce computation time drastically. Unlike most methods that assume a small error rate for observed genotypes (to cover genotyping errors, errors in the reference panel, mutations from the ancestral state, and gene conversions), HAPI-UR sets the probability of all reference haplotypes that disagree with the observed haplotype to 0. This allows the evaluation of which haplotypes fit an individual’s chromosome to be re-formulated as a bit-wise set-intersection operation. In addition to this, HAPI-UR uses a structured representation of the reference haplotypes that allows for fast lookups of matching haplotypes, and for each individual, it creates individual specific diploid HMM, which ignore all haplotypes that disagree with homozygous sites. Instead of using a fixed window size, HAPI-UR uses dynamic windows that begin with a small size (4 markers) and grow to a user specified maximum (e.g. 64 markers) allowing the method to capture longer chromosome segments.

Eagle2 takes a different approach to phasing individuals by not using a window-based haplotype representation. Instead, Eagle2 uses a highly efficient reference haplotype storage method based on the positional Burrows–Wheeler Transform [24] to allow for consistent haplotype pairs to be identified in constant time. Instead of using a full HMM to evaluate all possible haplotypes, Eagle2 employs a beam search to evaluate only the most promising paths in the space of all possible haplotype pairs. At each heterozygous locus, these paths branch into two possible sub-paths based on the two phasing options. Low probability paths are pruned or merged to keep the overall number of paths small. To decrease the impact that errors in one part of the genome have on subsequent paths, haplotypes are called after 20 markers, allowing for the back-propagation of relevant genetic information while decreasing the potential impact of genotyping errors. Absence of approximate window-based haplotype representation makes Eagle2 particularly appealing for breeding populations, where a large number of close relatives share long chromosome segments.

## Methods

We evaluated the performance of the four diploid imputation methods, fastPHASE, Beagle v4.0, IMPUTE2, and MaCH and the three phasing methods, SHAPEIT2, HAPI-UR, and Eagle2 followed by three haploid imputation methods, IMPUTE2, Beagle v4.1, and Minimac3 on a series of simulated datasets and a real dataset.

The simulated dataset modelled a cattle population. The population consisted of five generations of 2000 animals, genotyped for single nucleotide polymorphisms (SNPs) on a single chromosome. Each generation was produced by selecting 100 sires from the previous generation based on their true breeding values and mating them with 1000 dams at random. The initial set of haplotypes was sampled using a Markovian Coalescent Simulator [25], assuming a single 100-cM long chromosome that was simulated using a per site mutation rate of 2.5 × 10^−8^, and an effective population size (Ne) that changed over time. Based on estimates for the Holstein cattle population [26], Ne was set to 100 in the final generation of simulation and to 1256, 4350, and 43 500 at 1000, 10,000, and 100,000 generations ago, with linear changes in between. The simulation of breeding values and of haplotypes of progeny were performed using AlphaSim [27].

In the baseline scenario, a single chromosome was genotyped either with a high-density array of 1000 SNPs (allele frequency greater than 0.01) or with a low-density array of 200 SNPs, evenly spaced across the high-density array. These numbers of SNPs per chromosome are order equivalent to some of the commonly used arrays in livestock. To cover the full span of arrays used in livestock, we varied the number of high-density and low-density SNPs as described in the following section. All sires and 100 dams were genotyped with a high-density array, while the remaining animals were genotyped with a low-density array. Pedigree information was not used in any of the phasing or imputation algorithms. To test the robustness of each method we independently modified the baseline scenario by varying:

- the number of SNPs on the low-density array from 5 to 400,
- the number of individuals in the population from 200 to 10,000, randomly selecting individuals that were genotyped at both high- and low-density,
- the number of genotyped dams from 0 to 500,
- the number of SNPs on the high-density array from 3000 to 45,000, while keeping the ratio of low-density to high-density SNP constant at 15:1.

We also considered the case in which the first two generations were genotyped on a different high-density array than the next two generations, with either 25, 50, or 75% of the SNPs overlapping between the two high-density arrays.

To compare the methods on a real dataset, we performed imputation on 56,607 individuals from a commercial pig breeding program. These animals were genotyped either with a high-density array of 60,000 or 80,000 SNPs or with a low-density array of 15,000 SNPs. To estimate imputation accuracy, we selected 500 high-density animals (typed at 60,000 SNPs) and masked part of their genotypes to mimic the pattern of missingness found for 500 animals that were genotyped at low-density. We restricted imputation to chromosome 1.

Imputation accuracy was measured as the correlation between the animals’ imputed genotypes and their true genotypes for each animal separately and averaged over all animals. The genotypes were not centered or standardized prior to calculating the correlation. While not centering or standardizing the alleles may increase the resulting correlations, it also makes the correlations independent of the minor allele frequency. We did not assess phasing accuracy independently of imputation accuracy.

For the simulated datasets, each method was given 8 GB of memory and 24 h to run. Jobs were terminated if they exceeded the runtime or memory limits. Unless otherwise specified, we used the default parameters for each simulation. We tested IMPUTE2 using either the default 10-cM windows or the entire chromosome and found that imputing the entire chromosome increased accuracy at the cost of additional computational time. We used windows of 5 cM with an overlap of 1 cM for Beagle v4.0 and Beagle v4.1. The real dataset was only imputed using the two-step imputation methods, given their high accuracy and shorter runtimes.

In all cases, high-density and low-density individuals were phased separately. In the case of multiple high-density arrays, we used the “merge_ref_panels” option in IMPUTE2 and phased genotypes from the two high-density arrays separately. Because neither Minimac3 nor Beagle v4.1 accept multiple high-density arrays, we phased the high-density individuals together and let the phasing method fill in the missing genotypes for high-density individuals.

## Results

### Accuracy

**Fig. 1:**
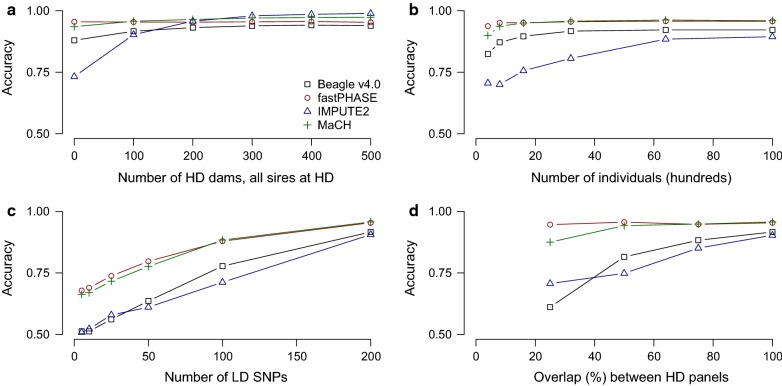
Genotype imputation accuracy of four diploid HMM algorithms based on simulated data. Unless otherwise noted, there were 1000 high-density (HD) SNPs per chromosome, 200 low-density (LD) SNPs per chromosome, 100 dams genotyped at high-density, and complete overlap between the high-density arrays of generations 1 and 2 and those of generations 3 and 4. We varied **a** the number of dams genotyped at high-density, **b** the number of individuals in the population, **c** the number of SNPs in the low-density array, and **d** the amount of overlap between the high-density array of generations 1 and 2 and those of generations 3 and 4

Performance of the diploid imputation methods is shown in Fig. 1. Among these methods, MaCH performed well in most settings. Its accuracy depended slightly on the number of high-density dams, the number of low-density SNPs, and the overlap between high-density arrays. The performance of fastPHASE was similar to that of MaCH but fastPHASE performed better when there was a small number of high-density animals or a small overlap between high-density arrays. Accuracies of IMPUTE2 and MaCH were similar but IMPUTE2 performed worse than MaCH when considering a small number of high-density dams, or a small number of individuals, and better when a large number of high-density dams was considered. Beagle v4.0 performed similarly to IMPUTE2 but was less affected by the number of high-density dams and the number of individuals. In all cases, the diploid imputation methods were not able to impute to more than 3000 SNPs per chromosome in 24 h and so they were not evaluated under that condition.

**Fig. 2:**
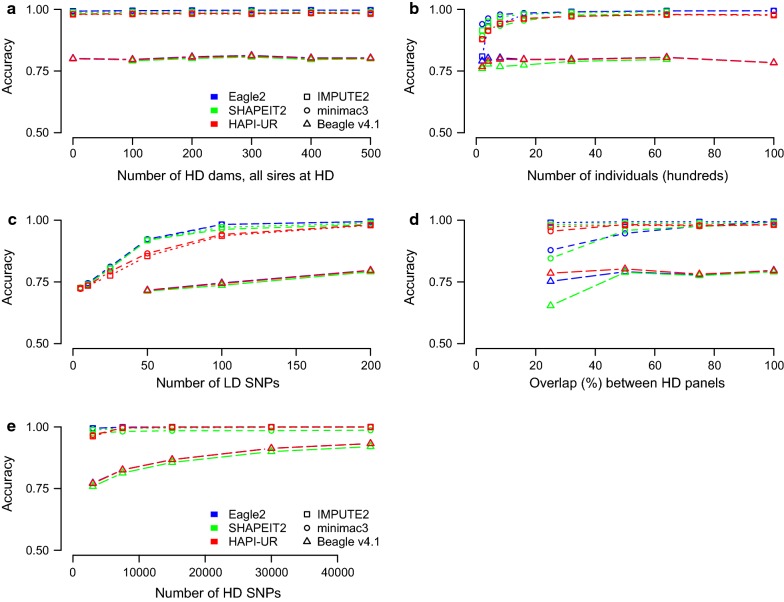
Genotype imputation accuracy of a combination of pre-phasing and haploid HMM methods based on simulated data. Unless otherwise noted there were 1000 high-density (HD) SNPs per chromosome, 200 low-density (LD) SNPs per chromosome, 100 dams genotyped at high-density and complete overlap between the high-density arrays of generations 1 and 2 and those of generations 3 and 4. We varied **a** the number of dams genotyped at high-density, **b** the number of individuals in the population, **c** the number of SNPs in the low-density array, **d** the amount of overlap between the high-density array of generations 1 and 2 and those of generations 3 and 4, and **e** the number of high density SNPs per chromosome keeping the ratio between high- and low-density constant (15:1)

Performance of the pre-phasing and haploid imputation methods is shown in Fig. 2. Among these methods, we found that the combination of Eagle2 and IMPUTE2 gave the highest imputation accuracy. Eagle2 led to the highest imputation accuracy regardless of the imputation method it was combined with, and led to higher accuracies than any of the diploid imputation methods. SHAPEIT2 led to a similar but slightly lower performance than Eagle2. HAPI-UR led to the lowest overall performance for low-density arrays but its performance increased as the number of high-density SNPs increased and reached the same accuracy as Eagle2 for arrays with more than 10,000 SNPs per chromosome. Of the tested haploid imputation methods, we found only a small difference between IMPUTE2 and Minimac3 but we found that Beaglev4.1 had poor imputation accuracy for all tested scenarios. We re-ran Beagle v4.1 with different-sized windows but did not see a noticeable increase in accuracy. The accuracy of Beaglev4.1 did increase as the total number of high-density SNPs increased but its accuracy was still substantially lower than that of either Minimac3 or IMPUTE2 at 45,000 SNPs per chromosome. There was no interaction between the choice of phasing method and the choice of imputation method for the overall imputation accuracy, except when multiple high-density arrays were used. In this case, the combination of HAPI-UR and Minimac3 outperformed the combination of Eagle2 and Minimac3.

### Run time and memory requirements

The elapsed run times for each method in the baseline scenario are in Table 1. Of the diploid imputation methods, MaCH had the shortest run time, followed by Beagle v4.0, fastPHASE, and IMPUTE2. Of the phasing methods, HAPI-UR was the fastest by an order of magnitude, followed by Eagle2 and SHAPEIT2. Of the haploid imputation methods, Minimac3 was the fastest, followed by Beagle v4.1 and IMPUTE2. The combined run-times of the two-step phasing and imputation methods were all substantially shorter than that of the single-step methods.

**Table 1:** Run time and accuracy for diploid imputation, phasing, and haploid imputation methods in the default simulated data scenario

Phasing method	Imputation method	Computing time (s)				Accuracy
		HD phasing	LD phasing	Imputation	Total	
	IMPUTE2			42,796	42,796	0.861
	Beagle v4.0			23,042	23,042	0.901
	MaCH			21,998	21,998	0.944
	fastPHASE			28,892	28,892	0.941
HAPI-UR	IMPUTE2	117	14	149	280	0.964
HAPI-UR	Minimac3	117	14	62	193	0.967
HAPI-UR	Beagle v4.1	117	14	78	209	0.793
Eagle2	IMPUTE2	1361	207	148	1717	0.988
Eagle2	Minimac3	1361	207	55	1623	0.988
Eagle2	Beagle v4.1	1361	207	79	1647	0.794
SHAPEIT2	IMPUTE2	8495	1175	150	9820	0.979
SHAPEIT2	Minimac3	8495	1175	58	9728	0.977
SHAPEIT2	Beagle v4.1	8495	1175	77	9747	0.792

The run time is given in seconds separately for phasing and imputation steps and as a total

*HD* high-density, *LD* low-density

### Real data

The two-step methods evaluated had similar performance on the real dataset (see Table 2). SHAPEIT2 was not able to phase the high-density and low-density individuals in 4 days and so was not analysed. Imputation accuracies of Eagle2 with Minimac3, Beagle v4.1, and IMPUTE2 were 0.992, 0.925 and 0.827, respectively. Imputation accuracies of HAPI-UR with Minimac3, Beagle v4.1, and IMPUTE2 were 0.995, 0.939 and 0.997%, respectively. Phasing with Eagle2 took 7 h distributed across eight cores. Phasing with HAPI-UR took 54 h on a single core. All haploid imputation methods took less than 6 h.

**Table 2:** Run time and accuracy for phasing, and haploid imputation methods on the real dataset scenario

Phasing method	Imputation method	Computing time (h)				Accuracy
		HD phasing	LD phasing	Imputation	Total	
HAPI-UR	IMPUTE2	11.53	43.09	5.63	60.25	0.997
HAPI-UR	Minimac3	11.53	43.09	2.27	56.89	0.995
HAPI-UR	Beagle v4.1	11.53	43.09	2.69	57.31	0.939
Eagle2	IMPUTE2	4.48 (8 cores)	2.37 (8 cores)	5.63	12.48	0.827
Eagle2	Minimac3	4.48 (8 cores)	2.37 (8 cores)	2.21	9.06	0.992
Eagle2	Beagle v4.1	4.48 (8 cores)	2.37 (8 cores)	4.19	11.04	0.925

The run time is given in hours separately for phasing and imputation steps and as a total. For Eagle2, the program was run distributed across 8 compute cores. HAPI-UR was run on a single core

*HD* high-density, *LD* low-density

## Discussion

In this paper, we evaluated the performance of HMM-based methods for imputation in animal breeding populations. We found that combinations of pre-phasing and haploid imputation methods led to greater imputation accuracy at substantially reduced runtimes compared to diploid imputation methods, even for a very small number of low-density markers or for a small number of high-density genotyped dams and small numbers of genotyped individuals. The combination of using Eagle2 to pre-phase genotypes and using Minimac3 for imputation led to high accuracy imputation in a wide range of simulation scenarios and when analysing a real animal population.

Our results highlight the power of phasing and imputing genotypes separately. Intuitively it makes sense that performing phasing and imputation in a single step may increase imputation accuracy by marginalizing over uncertainty in phasing. However, the results suggest that the additional accuracy lost by marginalizing over phasing errors is outweighed by the accuracy gained by considering larger haplotype reference panels. These results are particularly surprising in the context of animal breeding populations where pre-existing reference panels may not exist (at least in the public domain), and so the reference panel itself is inferred by phasing high-density genotyped individuals. Our results suggest that modern phasing methods have a sufficiently high accuracy for this phasing to lead to only a small number of imputation errors.

The superior performance of pre-phasing and haploid imputation is also surprising given the lower density of SNP arrays (both high-density and low-density) and the substantially smaller numbers of genotyped individuals that we used compared to recent human genetics studies. These results also suggest that when working with multiple low-density SNP arrays, individual SNP arrays can be phased and imputed separately.

Of the three phasing methods that we evaluated, Eagle2 led to the most accurate imputation, probably because it is able to exploit longer segments of shared haplotypes between individuals, which are very common in closely related breeding populations. Although Eagle2 resulted in the highest imputation accuracy, we found that HAPI-UR was an order of magnitude faster for most datasets and resulted in a small decrease in accuracy for the simulated scenarios but no decrease in accuracy for the real dataset. In their original paper, the authors of HAPI-UR suggest that it may be possible to increase the accuracy of HAPI-UR by running it multiple times with different window start positions and taking the consensus phase [19]. Due to the short run time, this strategy would be feasible in animal populations but was not analysed here. SHAPEIT2, the oldest of the phasing methods, had the longest run-time, which prevented us from evaluating it for the real dataset. Although the authors of SHAPEIT2 have now released SHAPEIT3, they do not recommend its use for populations with less than 60,000 individuals and so the performance of SHAPEIT3 was not analysed here. The great speed increases of HAPI-UR and Eagle2 over SHAPEIT2 are also notable because it closes the gap between HMM-based imputation methods and traditional heuristic imputation methods. For example, Miar et al. [2] found that, for phasing, FImpute was over a hundred times faster than SHAPEIT2. In this paper, we found that HAPI-UR was over fifty times faster than SHAPEIT2, which suggests that the difference between HAPI-UR and a heuristic pedigree-based method may be of a similar order of magnitude (a fact supported by pilot simulations, not shown).

We found little difference in performance between the assessed haploid imputation methods. Both Minimac3 and IMPUTE2 led to accurate imputation. The accuracy of IMPUTE2 was consistently slightly (< 1%) higher than that of Minimac3 with the simulated data but the runtime of IMPUTE2 was between two and three times longer than that of Minimac3. For the real dataset, the imputation accuracy of IMPUTE2 dropped when Eagle2 was used to pre-phase the data but remained high when HAPI-UR was used to pre-phase the data. Overall, the performance of Beagle v4.1 was poor in the context of the simulated datasets, although it improved in the context of the real dataset. As the number of SNPs increased, the performance of Beagle v4.1 also increased. This may be a result of the approximations used in Beagle v4.1, which were designed for imputation of human high-density SNP arrays to whole-genome sequence data and which may be less appropriate for the lower-density SNP arrays used in animal breeding populations.

With two exceptions, we found little interaction between the choice of phasing method and the choice of haploid imputation method. The first exception was observed for HAPI-UR when individuals were genotyped with multiple, semi-overlapping, SNP arrays, where the imputation accuracy obtained for HAPI-UR with Minimac3 or Beagle v4.1 was substantially higher than accuracy obtained for Eagle2 with Minimac3 or Beagle v4.1, although the accuracy of HAPI-UR with IMPUTE2 was lower than that of Eagle2 with IMPUTE2. The reason for this interaction stems from the fact that, in the case of Minimac3 and Beagle v4.1, the phasing algorithms were also used to perform imputation on the missing non-overlapping SNPs for each high-density array, whereas in IMPUTE2 the two high-density arrays were phased separately, and IMPUTE2 was used to fill in missing SNPs as part of its high-density array merging step. The higher accuracy obtained with HAPI-UR over Eagle2 in this scenario suggests that HAPI-UR can impute untyped loci in high-density arrays better than Eagle2. This is consistent with the second exception, which was that the imputation accuracy obtained with HAPI-UR was at least as high, as that obtained with Eagle2 when performing imputation on the real dataset. Animals in the real dataset were genotyped with two high-density arrays and two low-density arrays that both exhibited a number of randomly missing SNP genotypes. When using Eagle2 to phase individuals, IMPUTE2 and Beagle v4.1 resulted in markedly lower imputation accuracy, in particular compared to Minimac3. In contrast, when HAPI-UR was used to phase individuals, the performance of Minimac3, IMPUTE2 and Beagle v4.1 remained high, which suggests an advantage of using HAPI-UR over Eagle2 when individuals are genotyped on multiple arrays or when observing a large amount of random missing genotypes.

Some of the analysed phasing methods have an option to use pedigree information to improve phasing. Although these options were originally designed to help phase and impute parent–progeny trios [28], they can also be used for larger pedigrees [29]. Previous work on phasing and imputing animal populations showed that combining pedigree and linkage information can improve phasing and imputation accuracy [1, 2]. In this paper, we focused only on HMM-based methods that use linkage-disequilibrium information for phasing and imputation, as originally proposed by Li and Stephens [11]. SHAPEIT2 [29], Beagle v4.0 [28], and HAPI-UR [19] all provide options to use parent–progeny trio information. However, the top two performing methods in this study, Eagle2 and Minimac3, do not provide this option. It is likely that pedigree information can be included in these algorithms by pre-phasing and pre-imputing an individual’s genotypes based on the genotypes of the individual’s parents. This is similar to the approach of DuoHMM [29]. Future work is needed to analyse how HMM can use pedigree information to improve phasing and imputation, and to integrate this with the high-performance methods that were reviewed and tested here.

## Conclusions

Overall, this study suggests that modern hidden Markov model pre-phasing and haploid imputation methods can perform fast and accurate imputation of SNP genotypes in animal breeding populations of any size without the use of pedigree. This is particularly important given an interest in performing imputation and implementing genetic selection in non-traditional livestock populations without recorded pedigree, for which existing heuristic methods may be less appropriate, such as in developing countries or on multiplier and commercial farms. We noticed no disadvantage of using the two-step imputation approach even in cases of small populations, low-density SNP arrays, or multiple high-density arrays. Of the algorithms, we found that Eagle2 and HAPI-UR both reliably pre-phased the data and that IMPUTE2 and Minimac3 led to the highest imputation accuracy. However, we also noted a decreased accuracy when Eagle2 and IMPUTE2 were used to pre-phase and impute the data when animals were genotyped with semi-overlapping high-density SNP arrays. In this case, using Eagle2 with Minimac3 and HAPI-UR with IMPUTE2 or Minimac3 led to higher accuracy. Overall, our findings highlight the importance and feasibility of using HMM to perform imputation in animal breeding populations, even as the number of genotyped animals and genotyping densities increase.
